# Review on Lymph Node Metastases, Sentinel Lymph Node Biopsy, and Lymphadenectomy in Sarcoma

**DOI:** 10.3390/curroncol31010020

**Published:** 2024-01-05

**Authors:** Paulina Chmiel, Maria Krotewicz, Anna Szumera-Ciećkiewicz, Ewa Bartnik, Anna M. Czarnecka, Piotr Rutkowski

**Affiliations:** 1 Department of Soft Tissue/Bone Sarcoma and Melanoma, Maria Sklodowska-Curie National Research Institute of Oncology, 02-781 Warsaw, Poland; gp.chmiel@gmail.com (P.C.); maria.krotewicz@nio.gov.pl (M.K.); piotr.rutkowski@pib-nio.pl (P.R.); 2 Department of Pathology, Maria Sklodowska Curie National Research Institute of Oncology, 02-781 Warsaw, Poland; anna.szumera-cieckiewicz@pib-nio.pl; 3 Institute of Genetics and Biotechnology, Faculty of Biology, University of Warsaw, 02-106 Warsaw, Poland; ebartnik@igib.uw.edu.pl; 4 Institute of Biochemistry and Biophysics, Polish Academy of Sciences, 02-106 Warsaw, Poland

**Keywords:** lymph nodes, lymphadenectomy, sarcoma, soft tissue sarcoma, STS, metastasis, prognosis, treatment

## Abstract

Soft tissue sarcomas (STS) originating from connective tissue rarely affect the lymph nodes. However, involvement of lymph nodes in STS is an important aspect of prognosis and treatment. Currently, there is no consensus on the diagnosis and management of lymph node metastases in STS. The key risk factor for nodal involvement is the histological subtype of sarcoma. Radiological and pathological evaluation seems to be the most effective method of assessing lymph nodes in these neoplasms. Thus, sentinel lymph node biopsy (SLNB), which has been shown to be valuable in the management of melanoma or breast cancer, may also be a beneficial diagnostic option in some high-risk STS subtypes. This review summarizes data on the risk factors and clinical characteristics of lymph node involvement in STS. Possible management and therapeutic options are also discussed.

## 1. Introduction

Soft tissue sarcomas (STS) are a relatively infrequent group of tumors, with an incidence rate (IR) of 5 per 100,000, meeting the criteria of a rare tumor [1]. They account for ~1% of all malignancies; however, increased IRs have been reported. This increase can be particularly attributed to the aging population but also to increased detection [2,3,4,5]. STS are heterogeneous malignancies, arising from mesenchymal tissue, comprising at least 100 different histological subtypes, each with different morphological features, translating into a unique clinical course and therapeutic approach [6]. Most patients at the time of diagnosis will present only locally advanced disease, but 25–60% of these patients will have distant metastases [7,8,9,10].

Understanding of how tumor cells migrate to the lymph nodes is still poor. Two main theories have been proposed. The first, proposed by Halsted, states that the metastatic process is a cascade of tumor cells that spread through the lymph nodes to distant locations [11]. The second theory highlights the importance of cancer as a systemic disease, in which cancer cells spread throughout the body in an early phase of tumor growth [12,13]. In summary, there is still no consensus on whether lymph node metastases indicate regional aggressiveness of the tumor or whether they are a primary point of dissemination [14]. Taking into account the current state of knowledge, the only well-documented correlation is between lymph node spread, the histological type of neoplasm, and the prognosis of the patient [15,16]. The current basis for the prognosis in oncological patients is staging, which is possible, in the case of lymph nodes, due to sentinel lymph node biopsy (SLNB) and lymph node dissection (LND) [17,18]. SLNB is a minimally invasive procedure that uses lymphoscintigraphy and ultrasound to detect the sentinel node, which is removed and sent for histopathological examination [19]. LND involves radical removal of all lymph nodes from the drainage route of the neoplasm area [20].

Accurate lymph node evaluation is crucial when the diagnosis and management of neoplasms such as melanoma, breast cancer, or gastric cancer [21,22,23]. SLNB in breast cancer is particularly beneficial for breast cancer patients with clinically negative nodes, establishing itself as a standard across various disease stages [24]. In melanoma treatment, the assessment of local lymph nodes has been used successfully for 30 years, and SLNB is especially recommended for patients with intermediate-thickness melanoma (1–4 mm) [25,26]. Striking a balance between SLNB and LND avoids unnecessary procedures while identifying patients who will benefit the most from resection. However, no strong guidelines covering lymph node metastases (LNM) in STS have been proposed andtreatment often depends on the experience of the center [27]. This state-of-the--of-the-art may result, among other things, from the rare occurrence and lack of research confirming the effectiveness of node biopsy/resection. Therefore, the objective of this article is to review the most recent data on lymph node metastases in soft tissue sarcomas, in correlation with their histological subtype and clinical presentation. Furthermore, diagnostic and therapeutic options are discussed, particularly in relation to the patients’ prognosis. 

## 2. Lymph Node Metastasis Frequency in Selected Sarcomas

Metastases have been shown to develop mainly through blood vessels, STS rarely spreads through the lymph vessels and occurs as lymph node metastases (LNM). Lymphatic spread has been reported in 1.75 to 9% of patients, depending on the study [28,29,30,31,32,33,34,35]. The predilection of certain histological types of sarcomas to lymphogenous dissemination should be the starting point for the consideration of probable therapeutic implications. Some histological forms, such as clear cell sarcoma, angiosarcoma, rhabdomyosarcoma, and epithelioid sarcoma (which together form the acronym CARE), show a predilection for lymph node involvement [36]. The reported incidence of lymph node disease in these high-risk histologies is 25% on average, with the highest range up to 55% [29,34,37,38]. Nodal spread is one of the bestbetter-documented prognostic factors in the case of STS. Historically, the Fifth Edition of the TNM Classification of Malignant Tumors has classified the presence of metastases in the lymph nodes as stage IV of the disease [39]. Therefore, the eighth edition of the AJCC Cancer Staging Manual contains four main prognostic factors for STS [40]. Among these is the presence of metastases in the lymph nodes, coded as characteristic N1 (in local lymph node involvement) or M1 (in non-local lymph node involvement) that determines the prognosis and consequently the treatment of the patient. Furthermore, analysis of studies on the frequency of metastases and their impact on prognosis in high-risk subtypes can provide additional information for optimal clinical management in these cases. 

### 2.1. General Statement

It is difficult to unambiguously determine the involvement of lymph node metastasis in STS. Guidelines for diagnostic procedures within the lymph nodes have not yet been developed, mainly due to the diversity and rarity of these diseases. Distant metastases pave the path of the disease and directly influence the prognosis, and lymph nodes are often neglected [41]. In recent years, numerous retrospective studies have collected data on nodal involvement in STS. 

Mazeron and Suit, who showed an incidence rate of 5.9% for LNM, made one of the first attempts to assess the frequency of nodal disease, the and dependence on the histological subtype, and the potential for nodal metastasis were demonstrated only in high-grade sarcomas (G2/3) [34]. Subsequent studies confirmed these results, with the lowest incidence at 1.75%, and the highest reaching 9%. Data from these studies from 1993 to 2022 were grouped according to risk of lymphatic spread and histological type and are presented in Table 1 and Table 2. The two largest analyzes included a total of more than 100,000 patients from the largest databases, Surveillance, Epidemiology and End Results (SEER) and the National Cancer Data Base (NCDB). Both analyzes found LNM in approximately 3.5% of patients with characteristic histological predilection [32,33]. Lou et al. also took into account the risk of lymph node metastases depending on the histological subtype, proving that rhabdomyosarcoma and angiosarcoma pose the highest risk, as previously mentioned [33]. The results of the research on synovial sarcoma (SS) are still contradictory; some authors classify SS as a high-risk histological type, but the latest studies do not seem to confirm this [42]. The contradictory results of the study by Jacobs et al. show that lymph node metastases in synovial sarcoma occur in 4.2% of patients, which is not significantly higher than the general proportion for all soft tissue sarcomas [43]. Therefore, the historical acronym ‘SCARE’ has been replaced by the current acronym we use, ‘CARE’.

In addition to the incidence rates of nodal metastases and histological type predilection, the above-mentioned studies provide information on possible risk factors for nodal involvement, indicating at-risk patient groups. The relationship between grading and LNM has been the best documented. Histological characteristics of the tumor are scored according to the Fédération Nationale des Centers de Lutte Contre le Cancer (FNCLCC) classification system and tumors are classified as grade 1 (low), 2 (intermediate), or 3 (high) [50]. High-grade tumors are associated with a more aggressive course, earlier metastasis, and a worse prognosis [51]. Ultimately, to date, all research has confirmed that high-grade tumors, mostly defined as G2 and G3, have the highest risk of lymph node metastases [31,33,34,44]. For example, Daigler et al. noticed that all tumors in their study were high grade and that, furthermore, among the 28 patients involved, two had the lymph node tumor grading changed to a higher grade compared with the primary site [45]. This may indicate the potential influence of lymph nodes on the course of the disease, but more in-depth research is needed. These results confirm the close relationship between tumor histological aggressiveness and lymphatic spread, making the grade a prognostic factor for LNM, independent of other characteristics of the patient.

Additional factors such as the primary site of the tumor, the age of the patient, or the size of the tumor are discussed less frequently, and the data are less clear. Tumors >5 cm appear to have more frequent lymph node involvement than tumors <5 cm, with the best documented data for large tumors (more than 10 cm diameter), though there is no consensus on this factor [31,45,46,52]. Tumor site and invasion depth are hallmarks of the prognosis in STS but play a marginal role in LNM [53,54]. Johannesmeyer et al. concluded that tumors localized in the upper extremity and with deep tissue invasion had a higher rate of regional lymph node metastases [31]. However, this is information from small sample studies, which makes it difficult to draw general conclusions.

Lymph node metastases profoundly impact prognosis, categorizing patients as stage III or IV in the AJCC classification. Documented positive nonregional lymph nodes are treated as metastatic disease with a significantly poorer prognosis. Patients with metastatic disease generally exhibit low survival rates, with median overall survival (OS) ranging from 14 to 24 months, and 2-year OS rates of up to 40% [41,55,56]. Taking into account the improvement in the diagnosis and treatment of these patients, OS rates increased over the years from 20% to 40% [55]. The prognostic role of lymph node dissemination is evident, with isolated patients with regional LNM having a better prognosis than those with distant metastases. At the same time, when comparing patients with N0 and N1 features, lymphatic dissection is a negative prognostic marker in general, with a shorter OS of five years. According to the AJCC analysis, patients with isolated regional nodal involvement had a 5-year OS better than those with distant metastases—33.1% vs. 12.4%, respectively [57]. Survival rates further differentiate between patients with localized disease (68%), nodal disease (38%), and metastatic disease (18%) [33]. Ferguson et al. confirmed these results with a 5-year survival rate for patients with regional LNM of 59% and only 8% for patients with pulmonary metastases [58]. Furthermore, the timing of LNM detection influences the prognosis, with a 5-year survival of 71% when detected >8 months after diagnosis and 19% when detected <8 months, predicting a poor prognosis in early detection [37]. Moreover, nodal dissemination is associated with a worse prognosis than invading the nerve, vessel, or bone [52]. Due to these significant differences in prognosis for specific groups of patients and the frequency of LNM accompanying distant metastases, control of the disease at the localized level appears to be crucial. 

In the following, we have collected comprehensive information related to the high-risk histological subtypes of STS and their correlation with LNM and prognosis. 

### 2.2. Clear Cell Sarcoma

**Figure 1:**
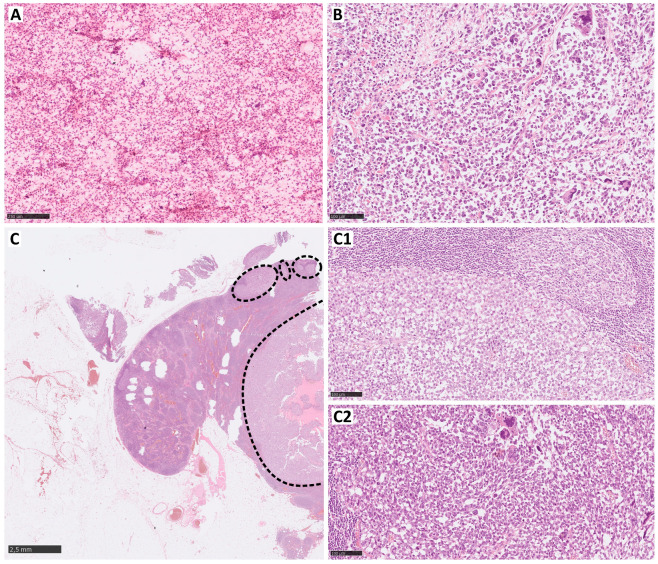
Clear cell sarcoma lymph multifocal metastasis (HE); the cytological image of the metastasis (**A**) and the morphology of the primary lesion (**B**) with the a majority of small round cells and scattered large pleomorphic cells. In the lymph node with multifocal, extensive metastases (**C**—dotted line), both morphological types of cells are seen (**C1**—small round cells and **C2**—pleomorphic, highly atypical).

Clear cell sarcoma (CCS) is one of the rarest sarcomas, with one of the highest rates of lymph node dissemination (Figure 1). The general 5-year survival in CCS is 62.9% and these values definitely differ between the localized disease and the regional spread with 5-year survival rates of 82.4% and 44%, respectively [59]. This observation indicates the importance of LNM in CCS. LNM rates in CCS vary depending on the study from 16 to even 39% [34,45,46]. Furthermore, clinical specialists are more strict about lymph node management in CCS, which is reflected in the increased willingness to perform lymphadenectomy and adjuvant radiation in the case of diagnosis [60]. Unlike epithelioid sarcoma, CCS has significantly worse median OS (23.8 vs. 49.6 months) but, compared with rhabdomyosarcoma, CCS patients have a better chance of prolonged survival [32,45]. However, the prognosis for these patients is serious, as indicated by a retrospective study that showed that all metastatic patients had died within a 5-year follow-up period [61]. 

### 2.3. Angiosarcoma

Angiosarcoma arises as a result of the neoplastic transformation of endothelial cells of blood or lymphatic vessels [62]. In this neoplasm, LNM occurs in approximately 20% of patients, which is relatively lower than in other CARE sarcomas [29,35,44]. The lowest prevalence observed was 8.1%, while the highest was 24%, similar to other high-risk subtypes [46,47]. Regardless of the lower incidence rate, angiosarcoma has a particularly unfavorable prognosis, especially when the lymph nodes are involved. The average 5-year survival value for these patients is up to 40% [62,63]. For patients with positive lymph nodes, these values drop to 22% [33]. Furthermore, the survival trends of patients with N1M0 disease more closely resemble those of M1 patients than those of non-metastatic patients [32]. In the case of scalp localization, more than 50% of patients have metastases in the lymph nodes and only 27.5% have distant metastases. The involvement of regional lymph nodes also predicts the occurrence of systemic disease within 3–6 months [64]. 

### 2.4. Rhabdomyosarcoma

Rhabdomyosarcoma (RMS) is one of the most common sarcomas in children, accounting for approximately 5% of malignancies in this age group [65]. Two main histological subtypes of RMS can be distinguished, embryonal (ERMS) in younger patients, located primarily in the head and neck or genitourinary tract, and alveolar (ARMS) in older patients, typically in locations of the extremities [66]. It is a rare malignancy in adults and is generally characterized by a more aggressive course, with 5-year OS for localized RMS and advanced RMS of 43% and 5%, respectively [67]. At the same time, in most studies, the pattern of lymph node metastases varies between these populations. In the pediatric population, lymph node involvement appears to be more common, appearing in up to 50% of cases [68,69]. Furthermore, among children, the ratio of positive lymph nodes to examined nodes is reflected in the prognosis. The higher the ratio, the worse the prognosis, with 10-year OS of 0% in cases where the ratio exceeded 0.75 [70]. In most studies in the adult population, LNM in rhabdomyosarcoma occurs in approximately 19–25% of patients [28,31,38,47]. Sherman et al. showed that, due to the high risk of metastases in the lymph nodes, rhabdomyosarcoma also has the highest rates of lymph node evaluation [46]. Some studies have stated that rhabdomyosarcoma has the highest frequency of lymph node metastases [30,47]. The primary tumor site and histological subtype of RMS can affect the presence of metastases in lymph nodes. Children studies in particular suggest a poorer prognosis for extremity RMS, due to alveolar differentiation and a significant rate of lymph node involvement [71]. Lymph node involvement also results in much more frequent diagnosis among patients with extremity RMS. The report of the EpSSG-RMS2005 study found that locoregional nodes are involved in 29% of patients in the pediatric group and that most of these patients undergo the SLNB procedure, which positively verified the initial stage [72]. In light of these data, pediatric RMS guidelines began to state the need for the surgical evaluation of lymph nodes, especially in patients diagnosed with ARMS, >10 years old, and with suspicious lymph nodes on imaging [73,74]. Sawamura et al. concluded that patients with other sarcoma subtypes had better OS than patients with rhabdomyosarcoma [35]. In the group of patients with RMS,, poor prognosis especially affects children and young adults with extremity alveolar RMS [75,76]. However, in adult patients there is no clear conclusion on this matter. 

### 2.5. Epithelioid Sarcoma

**Figure 2:**
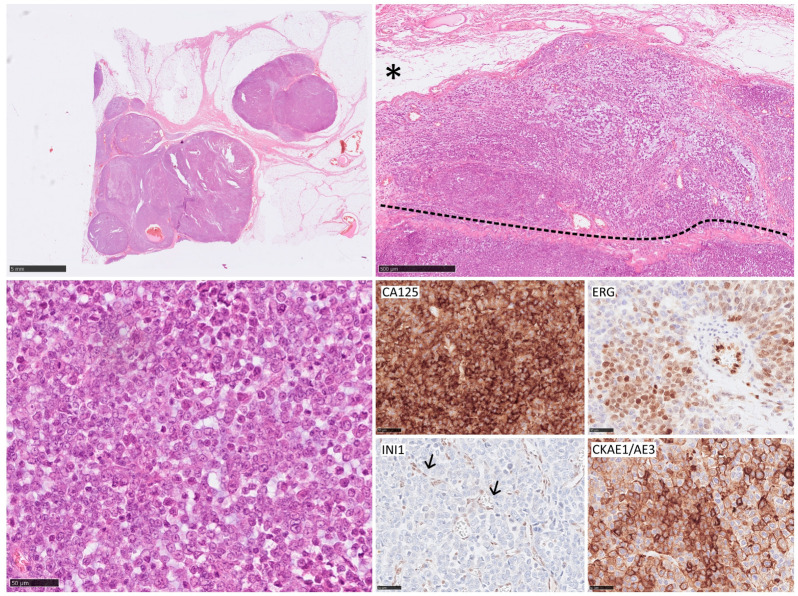
Lymph metastasis of epithelioid sarcoma and extension of the lymph node capsule (the dotted line) and invasion of fat tissue (asterix). Epithelial sarcoma typically expresses CA125, ERG, and cytokeratin (CKAE1/EA3). INI1 nuclear loss of INI1 expression is a characteristic feature (arrows—positive internal control in the endothelium).

Epithelioid sarcoma (ES) is a rather rare type of high-grade STS. It can be divided into proximal and distant types with different clinical courses and prognoses. Lymph node spread is common and can occur in approximately 30% of patients at the time of diagnosis (Figure 2) [77,78,79]. Of all high-risk sarcomas, ES appears to metastasize most frequently to the lymph nodes. The data provided are also the most consistent, with the least variation among the authors in the incidence of LNM. Of patients with ES, 17 to 31.8% will eventually develop LNM [28,29,35,38,45,46]. The established risk of LNM in ES is 13.4% (C.I. 95% 4.0–22.8) at 5 and 10 years. No differences have been observed between primary tumors and the recurrent group and all patients except one with lymphatic dissemination died after a median of 32 months [48]. Moreover, LNM are often diagnosed simultaneously with distant metastases [80]. It is not entirely clear whether the involvement of regional lymph nodes in ES should be treated as a disseminated disease, but studies have shown that only a small percentage of patients (one in four) with involved nodes will not develop distant metastases. However, nodal recurrences after radical resection are rare in ES [80]. Furthermore, no differences in results were observed when regional and distant nodes were compared [81]. The 5-year OS rates for ES were around 43.3%, a value that reduces in the presence of metastatic disease [82]. 

## 3. Diagnostic Assessment of Lymph Node Metastases in Sarcoma

Despite the significant impact on the course of the disease, STS management recommendations only briefly mention the evaluation of lymph nodes. Highly specialized sarcoma treatment centers often develop a consensus based on their experience [83,84]. Currently, there are many methods that can be used to assess the involvement of lymph nodes in the disease process, from the most basic clinical examination for local lymphadenopathy, through standard imaging techniques in cancer, such as ultrasound, CT, or magnetic resonance imaging, and and finally modern techniques, such as fluoro-2-deoxy-D-glucose (FDG)-PET [85]. However, their effectiveness and usefulness are low [86]. The final point of lymph node diagnostics should be SLNB or LND with pathological evaluation and IHC, which will clearly determine the presence and characteristics of metastases in the lymph nodes [87].

Lymph nodes in the cancer process may be clinically enlarged, but not necessarily metastatic [88]. Studies indicate that a large proportion of patients with clinically suspicious nodes who underwent a complete evaluation of the node had only benign reactive adenopathy and, in general, 1% had LNM [46]. Lymphadenopathy on clinical examination occurs in approximately 18% of STS patients, of whom only half had pathologically confirmed nodal metastases [89]. Keung et al. divided lymph node involvement into clinically suspicious but not pathologically confirmed (cN1) and pathologically confirmed (pN1). Among all evaluated patients, 55.5% had the characteristic cN1, but only 44.5% had confirmed nodal dissemination—pN1 [32]. Furthermore, complete nodal examination is performed mainly in high-risk CARE group sarcomas - most often in rhabdomyosarcoma (15.6%), angiosarcoma (10.0%), clear cell sarcoma (39.3%), or epithelioid sarcoma (28.1%) [46]. These findings suggest that lymph node metastasis may not be recognized during clinical examination, and that imaging techniques may provide an advantage in detection. The cornerstones of cross-sectional imaging used in lymph node assessment are CT and magnetic resonance imaging (MRI). The general rules for detecting cancer spread in the nodes can also be applied to STS. Research has shown that size and morphology in CT or magnetic resonance imaging are most significant when detecting pathology in lymph nodes [85]. Malignant lymph nodes are enlarged, round in shape, with a thick capsule and abnormal echogenicity, while benign lesions have a characteristic central fatty hilum, which is a distinctive feature when observing with CT and MRI [90]. Furthermore, metastatic nodes can have a signal enhancement pattern similar to that of the primary tumor [91]. However, clinical experience has shown that, in some cases, 90% of metastatic nodes were less than 1 cm and did not arouse suspicion on imaging [92]. The effectiveness of these methods is still debated, considering only the size of the nodes, magnetic resonance imaging has approximately 65% sensitivity and 70% specificity to detect malignant lymphadenopathy [93,94]. Adding distinct nodal characteristics, such as eccentric cortical hypertrophy or a long-to-short axis ratio, provides a better sensitivity and specificity of 79% and 93%, respectively [95]. Additionally, ultrasound (US) evaluation can be beneficial in diagnosis, especially for superficial lymph nodes and particularly in the head and neck or axilla [90]. The huge advantage of US is the possibility of obtaining an image-guided cytologic sample, with overall sensitivity and specificity of up to 95.45% and 98.25%, respectively [96]. However, MRI remains the preferred imaging modality to date [27].

18F-FDG PET/CT has been shown to represent a breakthrough in the imaging of lymph node metastases in STS, characterized by higher sensitivity compared with classic imaging methods. Multiple studies have shown the superiority of this method with a sensitivity of 86 to 100% and a specificity of 93 to 98% [97,98]. In rhabdomyosarcoma and angiosarcoma, correct interpretations of PET/CT were observed in 91.8% of the patients, and the N staging was correct in 97% of the patients [99]. Interestingly, a correlation was observed between the maximum standardized uptake value (SUVmax) and survival rates, with 5-year survival being 81% for patients with an SUVmax below 10 and 33% among patients with SUVmax >10 [97]. However, this method also has limitations, as it has been proven that the main false interpretations result from low uptake by small nodes containing micrometastases. Therefore, deposits smaller than 4–5 cm can be overlooked, and control imaging is beneficial for these patients [100,101]. In addition, the histological subtype affects the results and high-grade sarcomas, such as rhabdomyosarcoma, tend to show greater uptake of labeled glucose and are therefore more easily detected in PET/CT [102], in contrast myxoid liposarcoma or synovial sarcoma showed low glucose uptake and the usefulness of PET for these indications seems limited [86]. Organs with high physiological uptake of FDG, for instance the brain and the urinary bladder, can alter the detection of nodal involvement [87]. Inflammation or local processes that stimulate nodal uptake interfere with the results, producing more false positives [99]. The most recent method, not commonly used in clinical management, is PET with carbon-11 choline tracers (11C-choline), which allowed for the correct diagnosis in all patients with LNM, while conventional imaging accuracy was 63% [103]. These are usually primary tests, and an accurate determination of the stage N is performed during the pathological evaluation of the lymph nodes. In summary, whether the lymph nodes will be assessed in a full manner depends on the indicated tests and, therefore, any suspected lymph node detected clinically or radiologically is usually verified using SLNB or LND. 

## 4. SLNB

### 4.1. Technical Overview

**Figure 3:**
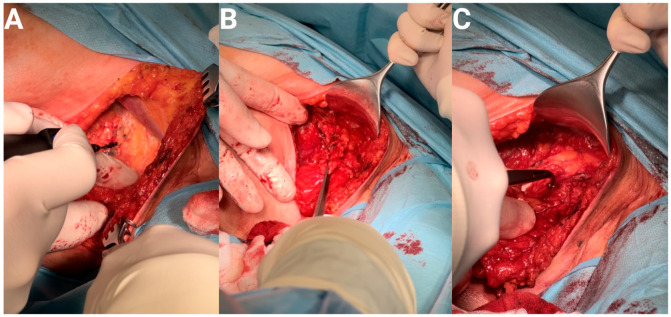
(**A**) Photograph of the beginning of axillary lymphadenectomy surgery. Patient with the diagnosis of rhabdomyosarcoma. (**B**) After administration of the patented blau dye. (**C**) After administration of the patented blau dye.

SLN biopsy is a minimally invasive procedure that allows the exclusion of the presence of sarcoma cells within the sentinel node. The need to perform SLNB is indicated in STS patients with a high risk of regional lymphatic spread, the CARE group, comprising clear cell sarcoma, angiosarcoma, rhabdomyosarcoma, and epithelioid sarcoma. The exclusion of the presence of sarcoma metastasis in SLN makes the absence of metastases in the remaining lymph nodes more likely and prevents LND. The sentinel node is identified using various types of tracers administered directly to the tumor site prior to SLNB. In our practice we detect sentinel nodes using the radioactive nuclide method and, for confirmation, the blue dye method (as described in the melanoma paper [104]). In the first of these methods radioactive tracer is administered to sarcoma tissue 24 h before SLNB. The sentinel node is the first node on the lymphatic flow path from the primary tumor lesion. Accumulated in the first lymph node, the sentinel node; the radioactive marker emits gamma rays. During the biopsy, a special gamma probe identifies the previously labeled node with the highest counts (Figure 3). The second method used when detecting sentinel nodes is a blue dye method known as the bioactive dye tracing method, which uses dyes such as patent blue, methylene blue and isosulfur blue [105]. This method is simple and cost effective. In order to mark the sentinel node, a suitable tracer is administered to the tumor area during the SLNB procedure, usually performed under general anesthesia. The blue dye diffuses and goes to lymphatic vessels and lymph nodes around the application site. The sentinel node is the first node stained with the dye and, after exposition, may be subjected to an excision biopsy. The biopsy of SLN detected by the above-mentioned methods is simple and in its typical course does not cause any subsequent complications; however, detection of the sentinel node is subject to the risk of lymphatic vessel leakage and allergic reaction to the applied tracer. Depending on the condition of the lymphatic vessels, more than one lymph node can be marked. Each of the marked nodes is considered as a sentinel node. The excised sentinel nodes are tested for the presence of sarcoma metastases. The accuracy of SLNB depends on the good cooperation of a multidisciplinary team, including a surgeon, nuclear medicine physician, and histopathologist.

### 4.2. Significance in STS

SLNB is accepted as a reasonable tool with which to help withof the prognosis of melanoma when the positive result rate is 10% or greater [106]. The SLNB positivity rates range from 4.3 to 50%, depending on the histological subtype and the study design and these differences are presented in Table 3. Applying this general recommendation, the use of SLNB in sarcoma should be considered in subtypes with high rates of lymph node metastases. In STS the data areare preliminary and sometimes contradictory. Thus far, no recommendations have mentioned performing SLNB even in the case of high-risk sarcomas [107,108]. Further prospective studies on the SLNB positivity rate in STS may help determine the optimal diagnostic and therapeutic approach. Most of the available analyses of the effectiveness of this procedure are conducted in a small group of selected patients with a high risk of bias, so interpretation should be cautious. Retrospective analysis of large databases of patients with STS indicates that SLNB is a rarely performed procedure. In most cases, it is used in high-risk subtypes such as rhabdomyosarcoma, CCS, and epithelioid sarcoma [109]. Many studies focus on the pediatric population with RMS. Of 537 patients with extremity RMS, nodal sampling was performed in 25.7% [110]. The largest meta-analysis on this topic showed that out, of 114 included patients, SLNB was positive in only 12%, while the estimated false negative rate from the meta-analysis was 29% [111]. Prospective studies appear to confirm this relationship, with a 12.9% incidence of SLNB positivity, which was most common in patients with CCS (6/12, 50%) followed by synovial sarcoma (2/42, 4.8%); no SLNB positivity was observed in patients with other STS subtypes. However, among 6 of 12 patients with positive SLNB and synovial sarcoma, only 2 had metastatic nodes in regional dissection [112]. In the pediatric population, the results correspond with those presented above; the SLNB positivity rate for patients with RMS was 16.7% (1/6) and 0% (0/17) for non-RMS sarcomas, giving an overall positivity rate of only 4.3% [113]. However, SLNB performed better than imaging studies. Thus, PET-CT has been found to suggest nodal involvement in many more patients than in those who had metastases on histopathological examination. An increase in FDG avidity was observed in the regional nodal basin in 14 of the patients (50%) and only 4 of those 14 patients were shown to have metastatic spread in the nodes. The positive predictive value of PET-CT was 29%, while the negative predictive value of PET-CT in this study was 79% [114]. Taking into account the diversity of STS and the difficulties in microscopic evaluation of tissue samples, maintaining a low false negative rate seems to be crucial for the usefulness of this procedure.

The prognostic value of SLNB is also controversial. For most studies, long-term results were interpreted mainly in terms of regional/distant recurrence-free survival (RFS) and OS. Excluding small trials that analyzed only a few patients, the studies have shown that OS outcomes in patients with positive SLN are worse than in patients with negative SLN [109,111,112,115]. Generally, patients with metastases detected in SLNB in the studies had an OS of 5 months, while negative patients had an OS of 48 months [97]. Patients with negative SLNB had improved overall survival (74% versus 40% at 5 years, *p* = 0.093), however, though the result was not statistically significant [112]. A similar trend was observed in the case of disease recurrence, regardless of the type of event. Patients with positive SLN had any relapse in 35.3%, local relapse in 5.9%, and distant metastases in 17.6%, while patients with negative biopsy had any relapse in 18.4%, local relapse in 7.9%, and distant metastasis in 2.6% [115]. However, in all of the mentioned studies, the 95% confidence intervals were wide, indicating the low quality of the data. This may be the reason why we also found studies with completely different results, in which patients with a positive SLNB had a 5-year OS of 71.4% compared with 71.9% for those with a negative SLNB [116]. Moreover, the authors of the a systematic review that collected studies on SLNB in STS did not draw any unambiguous conclusions. Among the six evaluated studies, half found no impact of SLNB on the course of the disease [87]. Furthermore, a comparison with the radiological assessment of lymph nodes showed that the tumor stage based on the SLNB classification has a prognostic value for the estimated OS, while there was no correlation for the radiological classification [115]. However, the prospective results proved that even a comprehensive assessment of lymph nodes and the implementation of adjuvant treatment, in patients with confirmed positive nodes, do not provide improved long-term outcomes in STS. Despite the application of optimal medical management, patients exhibited the onset of distant metastases, typically within 12 months. Furthermore, individuals with negative lymph node biopsy experienced the development of systemic disease during the follow-up period, involving the regional lymphatic drainage [112]. Therefore,, no correlation was found in the case of detection of nodal metastases at the initial diagnosis and at later stages of treatment.

## 5. LND

### 5.1. Technical Overview

The indication of LND is the presence of metastases in the sentinel nodes on histopathological examination confirmed by SLNB, especially for cases of N1M0 and N1M1 with distant resectable metastases. A qualification for LND should include an evaluation of the patient’s clinical condition and the results of laboratory tests, especially the serum lactic dehydrogenase test. Multiple unresectable distant sarcoma metastases imaged by CT or MRI exclude LND (30). LND is a surgical procedure for lymph node dissection performed using open surgery under anesthesia. A lymph node dissection can be regional or radical. In the regional LND some lymph nodes around the tumor are excised. The radical LND includes dissection of most or all lymph nodes in the tumor area. The area of treatment for LND depends on the location of the sarcoma. There are three main LND sites for sarcoma: axillary, inguinal, and cervical. In axillary LND, all nodes grouped at the low, mid, and high axillary level with the connective tissue capsules surrounding them are removed. The inguinal LND procedure includes lymph nodes located in the femoral triangle and deep inguinal nodes [117]. In cervical LND, as a rule, superficial and deep lymph nodes are dissected. The postoperative complications of LND depend on its location, type of surgery, and patient condition. Among the complications of LND, wound infection, wound necrosis, pain, seroma, hemorrhage, lymphedema, fistula, nerve damage, or respiratory distress can be mentioned.

### 5.2. Significance in STS

Radial lymphadenectomy has been the standard of care for regional lymph node metastases confirmed by biopsy [27,108]. Despite this, there is no consensus on the real utility of this procedure for patient outcomes and most studies are contradictory. Unfortunately, most of the studies retrospectively evaluated lymphadenectomy, and patients received other therapies as part of their treatment regimens, including external beam radiation, systemic chemotherapy, and, in certain cases, isolated limb perfusion. Several investigators observed benefits to prolonged survival after radical lymphadenectomy in patients with metastases confirmed by SLNB [29,38,87,112]. Furthermore, the palliative use of LND turned out to be beneficial to the quality of life in most patients but did not affect overall survival [44,45]. Comparing the treatment results of patients who underwent radical lymphadenectomy with those who underwent the procedure non-radically or did not undergo the procedure showed a significant improvement in survival. In all groups with high-risk STS subtypes, overall and cancer-specific survival rates were higher; however, this was not observed in the case of patients with leiomyosarcoma [118]. In addition, complete omission of regional lymph node evaluation was associated with a higher risk of death, especially in patients with epithelioid and clear cell sarcoma [119]. Nevertheless, other studies do not confirm these results. In most cases, the obtained results are not statistically significant when comparing patients who underwent lymphadenectomy. Sawamura et al. have stated that LND benefit was lost beyond two years of observation, leading to the conclusion that surgery has no significant long-term effect on outcome [35]. Similarly, other studies have indicated that the extent of lymph node resection did not affect survival among node-positive patients [31,120,121]. No differences in survival were found between patients who underwent LND and those who received non-operative treatment 5 years after surgery, with a respective 38% vs. 34% (*p* = 0.832) [120]. However, in clinical practice, even isolated lymph node metastases require aggressive treatment. As mentioned above, the consensus on this matter is based on expert opinions and procedures may differ between clinical centers. In such cases, depending on the subtype of sarcoma, and in addition to LND, RT with or without ChT is used. According to the guidelines, neoadjuvant radiation therapy and chemotherapy can be used for sensitive histological types. Preoperative chemotherapy is also available. In the case of patients with a large number of positive lymph nodes, in addition to LND, postoperative radiotherapy can be administered to the site after node resection [108]. In addition, less popular methods, such as regional hyperthermia, in addition to systemic ChT and isolated limb perfusion, have shown benefits in these cases [122].

## 6. Conclusions

The occurrence of LNM is primarily associated with an unfavorable outcome in STS. Therefore, clinicians are continuously striving to develop efficient management for these patients. Lymphatic dissemination is rare in the natural course of most STS; however, there is a group of high-risk ‘CARE’ sarcomas with a dominant predilection for the lymph nodes. In these subtypes, LNM have shown a significant impact on the prognosis and the spread of cancer, often being a proof of systemic disease rather than local advancement. SLNB has been proven to be a minimally invasive and beneficial diagnostic tool in breast cancer and melanoma. However, studies on its use in STS are scarce and lack strong evidence. The current results are contradictory and the positive rates of SLNB vary significantly depending on the study. Thus, a minority of the reviewed studies achieved positivity rates of 10%. There is no standardized technique for performing SLNB in STS, which limits the rigor and methodology of the cited research; also, and no validation can be performed. Furthermore, the role of the intraoperative pathological exam in SLNB is not ambiguously established. In our experience real-time assessment may be useful only in a prognostic manner. In particular, there are no studies evaluating the accuracy of SLNB and providing the false negative rate in STS, as has been extensively studied in the treatment of breast cancer and melanoma. 

In conclusion, we have a sufficient number of small retrospective studies with unclear methodology, but lack prospective validation and confirmation of results in large clinical trials that could confirm the value of performing a lymph node biopsy. It appears that, due to the percentage of lymph node metastases in high-risk CARE subtypes, their examination is beneficial, though it is still unknown what the optimal treatment would be. Furthermore, the variability in STS management poses a significant challenge in understanding the impact of SLNB. Most STS cases are treated outside of specialized sarcoma centers, without a multidisciplinary approach. Even among experts in sarcoma centers, there are variations in STS management. This is apparent in the the selection bias and the lack oflack of standardization in the offered techniques. As a result, numerous questions regarding regional lymph node metastasis in STS remain unsolved. The true incidence and prevalence of nodal metastasis for each sarcoma subtype are unknown due to the absence of standardized diagnostic approaches. The prognostic impact of LNM remains unclear and the survival benefit of resecting LNM, whether through SLNB or radical lymphadenectomy, has yet to be established.

## Figures and Tables

**Table 1:** Lymph node metastasis frequency in sarcomas from the ‘CARE’ group.

Study	Reference	Lymph Node Involvement—All Subtypes	Clear Cell Sarcoma	Angiosarcoma	Rhabdomyosarcoma	Epithelioid Sarcoma
Sawamura et al.	[35]	6% (49/87)	38% (3/8)	20% (2/10)	37% (7/19)	30% (6/20)
Johannesmeyer et al.	[31]	0.9% (64/7159)	11% (9/70)	N/A *	9.7% (11/113)	13% (15/155)
Mazeron et al.	[34]	5.9% (19/323)	N/A	(2/5)	(5/14)	4/5
Gandhi et al.	[30]	5.8% (21/326)	(2/19) 10.5%	N/A	31.5% (8/19)	N/A
Basile et al.	[28]	4.5% (120/2689)	27.6%	14.0%	17.3%	21.9%
Keung et al.	[32]	3.5% (3154/89,870)	15.9%	6.1%	N/A	13.1%
Fong et al.	[29]	2.6% (46/1772)	N/A	13.5% (5/37)	13.6% (12/88)	16.7% (2/12)
Liu et al.	[33]	6.02% (1081/17,937)	N/A	15.43%	26.88%	N/A
Riad et al.	[38]	3.7% (39/1066)	11.1% (2/18)	11.1% (2/18)	19% (4/21)	20% (3/15)
Jacobs et al.	[43]	5.3% (820/15,525)	N/A	N/A	N/A	N/A
Behranwala et al.	[44]	3.4% (73/2127)	4% (1/25)	10.9% (5/46)	22.2% (12/54)	18.5% (5/27)
Daigeler et al.	[45]	1.75%	17.6%	7.9%	6.0%	21.4%
Sherman et al.	[46]	1.05%	27.7% (28/290)	32.1% (17/290)	24.1% (14/290)	31.8% (34/290)
Gusho et al.	[47]	3.7% (547/1936)	18.8% (24/547)	26.7% (105/547)	8.1% (19/547)	14.5% (32/547)
Sambri et al.	[48]	N/A	N/A	N/A	N/A	19% (15/77)

* N/A—not available.

**Table 2:** Lymph node metastasis frequency in non-high risk sarcoma histological subtypes.

Study	Reference	Lymph Node Involvement—All Subtypes	Synovial Sarcoma	Leiomyosarcoma	Liposarcoma	Myxofibrosarcoma	Malignant Peripheral Nerve Sheath Tumor (MPNST)
Sawamura et al.	[35]	6% (49/87)	N/A	N/A	N/A	N/A	N/A
Johannesmeyer et al.	[31]	0.9% (64/7159)	N/A	N/A	N/A	N/A	N/A
Mazeron et al.	[34]	5.9% (19/323)	0/4	1/10	N/A	N/A	N/A
Gandhi et al.	[30]	5.8% (21/326)	10.5% (2/19)	N/A	N/A	N/A	10.5% (2/19)
Basile et al.	[28]	4.5% (120/2689)	2.7%	3.1%	N/A	5.2% (11/212)	5.1% (7/138)
Keung et al.	[32]	3.5% (3154/8970)	3.3%	3.0%	1.6%	1.6%	3.8%
Fong et al.	[29]	2.6% (46/1772)	N/A	N/A	N/A	N/A	N/A
Liu et al.	[33]	6.02% (1081/17,937)	5.23%	5.08%	3.92% (190/5105)	N/A	6.25% (1/16)
Riad et al.	[38]	3.7% (39/1066)	N/A	N/A	2.4% (6/249)	N/A	4.9% (2/41)
Jacobs et al.	[43]	5.3% (820/15,525)	4.2%	N/A	N/A	N/A	N/A
Behranwala et al.	[44]	3.4% (73/2127)	4.1% (7/171)	2.7% (13/483)	0.9% (3/340)	N/A	4.2% (4/95)
Daigeler et al.	[45]	1.75%	0.6%	3.6%	0.3%	N/A	3.2%
Sherman et al.	[46]	1.05%	6.0% (9/290)	7.5% (14/290)	N/A	N/A	N/A
Gusho et al.	[47]	3.7% (547/1936)	3.2% (31/547)	1.4% (22/547)	N/A	N/A	N/A
Sambri et al.	[49]	N/A	N/A	N/A	N/A	3.9% (5/128)	N/A

**Table 3:** Rates of SLNB positivity in different studies for STS.

Tumor Type	Positive/Total SLNB (%)	References
Rhabdomyosarcoma	7.7%, 16.7%, 23%, 42.8%	[111,113,114,115]
Epithelioid sarcoma	0%, 9.8%, 14.2%	[113,114,115]
Clear cell sarcoma	0%, 10.8%, 14.2%, 35%, 50%	[111,112,113,114,115]
Angiosarcoma	3.2%	[115]
Leiomyosarcoma	1.1%	[115]
Synovial sarcoma	0%, 1.5%, 4.3%, 6%	[111,112,113,115]
